# Thermal, Rheological, Mechanical, and Electrical Properties of Polypropylene/Multi-Walled Carbon Nanotube Nanocomposites

**DOI:** 10.3390/polym13020187

**Published:** 2021-01-07

**Authors:** Nicoleta-Violeta Stanciu, Felicia Stan, Ionut-Laurentiu Sandu, Catalin Fetecau, Adriana-Madalina Turcanu

**Affiliations:** Center of Excellence Polymer Processing, Dunarea de Jos University of Galati, 47 Domneasca, 800 008 Galati, Romania; nicoleta.stanciu@ugal.ro (N.-V.S.); laurentiu.sandu@ugal.ro (I.-L.S.); catalin.fetecau@ugal.ro (C.F.); madalina.constantinescu@ugal.ro (A.-M.T.)

**Keywords:** polypropylene, carbon nanotubes, viscosity, specific volume, thermal conductivity, electrical conductivity, mechanical properties, injection molding

## Abstract

In this paper, nanocomposites based on polypropylene (PP) filled with up to 5 wt.% of multi-walled carbon nanotubes (MWCNTs) were investigated for determining the material property data used in numerical simulation of manufacturing processes such as the injection molding and extrusion. PP/MWCNT nanocomposite pellets were characterized for rheological behavior, crystallinity, specific volume and thermal conductivity, while injection-molded samples were characterized for mechanical and electrical properties. The addition of MWCNTs does not significantly change the melting and crystallization behavior of the PP/MWCNT nanocomposites. The effect of MWCNTs on melt shear viscosity is more pronounced at low shear rates and MWCNT loadings of 1–5 wt.%. However, with the addition of up to 5 wt.% of MWCNTs, the PP/MWCNT nanocomposite still behaves like a non-Newtonian fluid. The specific volume of the PP/MWCNT nanocomposites decreases with increasing MWCNT loading, especially in the MWCNT range of 1–5 wt.%, indicating better dimensional stability. The thermal conductivity, depending on the pressure, MWCNT wt.% and temperature, did not exceed 0.35 W/m·K. The PP/MWCNT nanocomposite is electrical non-conductive up to 3 wt.%, whereas after the percolating path is created, the nanocomposite with 5 wt.% becomes semi-conductive with an electrical conductivity of 10^−1^ S/m. The tensile modulus, tensile strength and stress at break increase with increasing MWCNT loading, whereas the elongation at break significantly decreases with increasing MWCNT loading. The Cross and modified 2-domain Tait models are suitable for predicting the melt shear viscosity and specific volume as a function of MWCNTs, respectively. These results enable users to integrate the PP/MWCNT nanocomposites into computer aided engineering analysis.

## 1. Introduction

In the field of nanocomposites, polymer/carbon nanotube (CNT) nanocomposites have attracted tremendous research and industrial interest owing to the CNTs outstanding electrical conductivity [1,2,3,4], thermal conductivity [4,5] and mechanical properties [4,6,7,8]. Moreover, CNTs show great potential for electromagnetic interface shielding and noise reduction [9,10]. In this context, polymer/CNT nanocomposites with tailored mechanical, electrical or thermal properties have been designed for different applications concerning aerospace, electronic and electromagnetic or automotive fields, among a few [4,8,11], leading to an increased number of demands for integrating the polymer/CNT nanocomposites within computer aided engineering (CAE) and finite element analysis (FEA).

Injection molding is the most widely used manufacturing process for mass-production of polymers and polymer-based composites [12,13,14,15,16] and, as the molded parts grow in complexity, numerical simulation is commonly used to optimize the injection molding process in terms of part geometry (e.g., design and prototype of molds/tools) and process parameters [13,14,15,16], and to predict potential defects such as air traps, weld lines, sink marks, warpage and shrinkage [13,14,15,16,17,18,19,20].

In general, the accuracy of the numerical simulations is not associated with the simulation models but rather with uncertainties in the input material parameters. Therefore, reliable numerical simulation of the melt manufacturing processes (e.g., injection molding and extrusion) needs reliable material information (e.g., physical, mechanical, and rheological properties, and specific volume) for each specific grade. Although, in the last years, numerous investigations have been conducted on characterization of polymer/CNT nanocomposites, the literature review has shown that there are no studies that provide a single source of materials property data. Therefore, data needed for numerical simulation of polymer/CNT nanocomposites may be obtained by combining the values of physical, thermal, and mechanical properties from different sources or selecting an equivalent material taking into account material family, filler content, melt flow rate, viscosity index, transition temperature, etc. However, this may lead to very serious errors since the material properties of polymer/CNT nanocomposites are influenced by many factors, including polymer matrix, type of CNTs, aspect ratio, dispersion and alignment of CNTs within the polymer matrix, processing methods, etc. [4,7,8,11,21]. In addition, the measurement methods can produce unreliable results, especially when anisotropic properties such as thermal [22,23,24] and electrical conductivity [24,25,26] are measured, which depend on the filler orientation during the manufacturing processes, including injection molding [22,23].

Polypropylene (PP) is one of the most versatile thermoplastics used for manufacturing of a variety of products due to its unique features and qualities, including good processability, high tensile strength, low density, high resistance to corrosion, chemical stability, and high impermeability [27,28,29,30,31,32]. Moreover, PP/CNT nanocomposites have received extensive attention in the last years. The addition of CNTs into PP results in nanocomposites with changed properties; some of them are advantageous (superior mechanical properties-improved Young modulus and tensile strength [27,30,31,32,33,34,35,36], higher heat deflection, higher thermal [30,37,38] and electrical conductivity [38,39,40,41,42,43,44,45,46,47], better warpage and shrinkage [33,48]), while others are less favorable (increased viscosity [29,31,39,40,42,44,49,50,51] or decreased ductility [30,31,32,33,34,35]).

Numerous research papers investigated different aspects of the structure–property–relationships of PP/CNT nanocomposites [27,28,29,30,31,32,33,34,35,36,37,38,39,40,41,42,43,44,45,46,47,48,49,50,51]. However, as already underlined, less attention has been dedicated to investigate the materials property data that can be used for numerical simulation of PP/CNT nanocomposite manufacturing processes. Therefore, the aim of this work is to provide a single source of materials property data for engineering calculations and simulations of melt manufacturing processes such as injection molding and extrusion. In particular, the rheological, thermal, mechanical, and electrical properties and the specific volume of the PP/MWCNT nanocomposites (0.1, 0.3, 0.5, 1, 3, and 5 wt.%) were investigated. Injection molding was used for the preparation of PP/MWCNT nanocomposite samples for mechanical and electrical characterization. In addition, the experimental results were supplemented by theoretical calculations on melt shear viscosity and specific volume.

## 2. Materials and Methods

### 2.1. Materials

The nanocomposites under investigation are based on industrial PP2001 masterbatch (Nanocyl S.A., Sambreville, Belgium) with 20 wt.% of multi-walled carbon nanotubes (Nanocyl NC7000, Nanocyl S.A., Sambreville, Belgium) produced via catalytic carbon vapor deposition (CCVD) process [52]. PP/MWCNT pellets with 0.1, 0.3, 0.5, 1, 3, and 5 wt.% of MWCNTs were purchased from Nanocyl. According to the supplier, the PP2001 masterbatch [53] was diluted with PP Moplen HP 400R (LyondellBasell Industries Holdings B.V., Rotterdam, The Netherlands) by melt-mixing at a melt temperature of 190 °C. As reported by the manufacturer, the melt flow rate of the PP is 25 g/10 min (230 °C, 2.16 kg), and the density is 900 kg/m^3^ at room temperature [54].

### 2.2. Injection Molding

**Figure 1:**
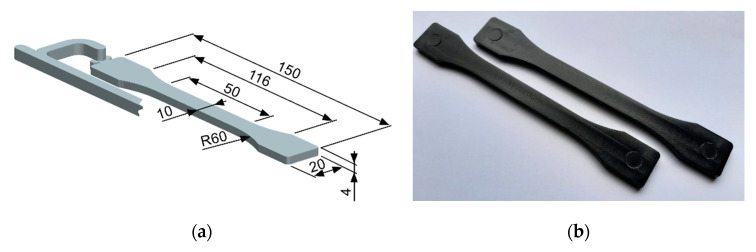
Geometry and dimensions (in mm) of the specimen (**a**) and injection-molded PP/MWCNT nanocomposite specimens (**b**).

Specimens according to ISO 527 were injection-molded on an Allrounder 320 C 500–170 injection molding machine (Arburg GmbH + Co KG, Lossburg, Germany). The geometry and dimensions of the specimen are shown in Figure 1a. The PP/MWCNT pellets were first dried at 90 °C for 2 h into a thermolift (Arburg GmbH + Co KG, Lossburg, Germany) and then conveyed to the injection molding machine. The injection molding experiments were carried out with the following parameters: injection flow rate, 30 cm^3^/s; maximum injection pressure, 500 bar; mold temperature, 50 °C; two melt temperatures (die zone), 200 and 220 °C; cooling time, 20 s. After injection molding, the specimens were conditioned in a standard laboratory environment for 24 h before further characterization. Figure 1b shows the injection-molded PP/MWCNT specimens with 5 wt.%.

### 2.3. Characterization

#### 2.3.1. Bulk Density

The bulk density of the PP/MWCNT nanocomposites was measured according to ISO 1183-1 using the Archimedes principle on an analytical balance (AB204-S/FACT, Mettler Toledo, Columbus, OH, USA) equipped with a kit density. The PP/MWCNT pellets were weighted both in air and ethanol, at room temperature, and the reported results are the averages of ten measurements.

#### 2.3.2. Differential Scanning Calorimetry (DSC)

The crystallization and melting temperatures of the PP/MWCNT nanocomposites were investigated using the DSC analysis performed on a DSC 200 F3 Maia^®^ (NETZSCH-Gerätebau GmbH, Selb, Germany) differential scanning calorimeter. A heating-cooling-heating cycle between 50 °C to 250 °C at a rate of 10 °C/min in N_2_ atmosphere (50 mL/min) was applied. The apparent enthalpies of fusion were calculated from the area under the endothermic peaks. The melting temperature (*T_m_*) was obtained from the endothermic peaks of the second run curves, whereas the crystallization temperature (*T*_c_) was determined from the peak of the first exothermic curve. The degree of crystallization was calculated by the following equation

(1) $$\chi (\%)=\frac{\Delta H_{m}}{\Delta H_{0}\cdot (1-\varphi )}\cdot 100\%,$$

where $\Delta H_{m}$ is the melt enthalpy of the sample, $\Delta H_{0}$ is the melting enthalpy of 100% crystalline PP (209 J/g for PP [55,56]) and φ is the weight fraction of MWCNTs.

#### 2.3.3. Melt Flow Index (MFI)

MFI measurements were performed based on the ISO 1133, using the CEAST Melt Flow Quick Index (Instron, Norwood, MA, USA) at 190 °C and 230 °C, under a load of 2.16 kg.

#### 2.3.4. Rheological Properties

The melt flow behavior of the PP/MWCNT nanocomposites was investigated under the capillary shear flow using a high-pressure capillary rheometer (Rheograph 75, Göttfert GmbH, Buchen, Germany) with three round capillary dies with length/diameter ratio of 30/1, 20/1, 10/1 and 180° entrance angle. The rheological measurements were carried out in the temperature range of 190–230 °C and shear rate range of 10–10,000 s^−1^. During the experiments, the shear rate was applied in decreasing order. Prior to rheological tests, the pellets were dried in a vacuum oven (EV-50, Raypa, Terrassa, Spain) at 80 °C for 4 h. To assess the stability of the nanocomposite melts under constant shear rate, tests were run at 220 °C on a 20/1 capillary die at constant shear rate of 250 s^−1^ for 2000 s.

It should be noted that, the Bagley correction (i.e., correction for pressure loss due to the reduction in radius between the barrel and the capillary) and Weißenberg-Rabinowitsch correction (i.e., correction for non-parabolic velocity profile) were applied to the capillary flow data to determine the real shear rate and melt shear viscosity. The calculations were carried out using the WinRHEO II software (Göttfert GmbH, Buchen, Germany). A more in-depth discussion on the correction procedures can be found in paper [57].

#### 2.3.5. Thermal Conductivity (TC)

Thermal conductivity measurements were carried out on a capillary rheometer (Rheograph 75, Göttfert GmbH, Buchen, Germany) equipped with a capillary quick-lock-system and a thermal conductivity probe. The measurements were carried out according to ASTM D5930 under cooling mode, in the temperature range of 200–70 °C, at 10 °C intervals. For each temperature, three pressures were applied in increasing order, i.e., 100, 250, and 500 bar, and, for each pressure, two successive measurements were run to check the reproducibility. Additional details on the measurement procedure can be found in [58]. Given the cylindrical shape of the sample, the TC was measured in radial direction and, therefore, is regarded as the through-plane TC. On the other hand, the preparation of the sample could be compared with compression molding process, where little flow of the molten matrix takes place, thus no significant filler orientation occurs, resulting in an isotropic material [22]. Therefore, the TC value might also be regarded as the average of thermal conductivity in the in-plane and through-plane directions.

#### 2.3.6. Specific Volume

A capillary rheometer (Rheograph 75, Göttfert GmbH, Buchen, Germany) equipped with a capillary quick-lock-system was used to measure the specific volume as a function of pressure and temperature (e.g., the *pVT* diagrams) The measurements were carried out according to ISO 17744, and additional details on the measurement principle can be found in [58,59]. The tests were carried out in cooling mode in order of decreasing temperature from 220 to 80 °C. For each temperature, the initial pressure was 10 bar, which was increased to 100, 250, 500, 750, 1000, and 1250 bar.

#### 2.3.7. Mechanical Properties

The tensile properties of the injection-molded PP/MWCNT specimens were measured using a M350–5AT Testometric machine (Testometric Co. Ltd., Rochdale, UK) with a 5 kN load cell. Tensile tests were performed at room temperature (23 ± 2 °C) at crosshead rates of 50, 100, and 500 mm/min and 115 mm gauge length. The mechanical properties were extracted from the engineering stress-strain curves according to ISO 527. For each nanotube wt.%, at least five specimens were tested and the reported values represents the average of five measurements.

#### 2.3.8. Electrical Conductivity

The two-point direct current (DC) method with reversal polarity was applied to determine the electrical conductivity of PP/MWCNT injection-molded samples [25]. The electrical resistance was measured with a DC power source (Keysight B2961A, Keysight Technologies, Santa Rosa, CA, USA) at a voltage level of +/−200V in longitudinal direction. To minimize the contact resistance, the injection-molded samples were coated with graphite conductive paint. For each nanotube loading, the electrical resistance measurement was carried out on five different specimens and the average value was used to calculate the resistance of the sample by Equation (2) [25]

(2) $$R=2\frac{R_{+}\cdot R_{-}}{\left(R_{+}+R_{-}\right)}\;(\Omega ),$$

in which $R_{+}$ and $R_{-}$ are the average equivalent resistance of the circuit under positive and negative voltage, respectively.

The volume resistivity of the sample was calculated by Equation (3)

(3) $$\rho =R\frac{S}{L}\;(\Omega \;m),$$

where S is the sample cross-section area perpendicular to current flow, L is the distance between the electrodes (e.g., 30 mm) and R is the electrical resistance of the composite specimens.

The electrical conductivity, σ (S/m), was calculated as the reciprocal (inverse) of volume resistivity.

### 2.4. Statistical Analysis

Normality of the data distribution from mechanical and electrical measurements was tested with the Shapiro-Wilk test. In case of normal distributions, statistical differences between two groups were investigated with a 2-sample *t*-test. The effect of input parameters was investigated with the Analysis of Variance (ANOVA). All statistical analyses were performed with the Minitab^®^ 19 software (State College, PA, USA) at a significance level of 5%.

## 3. Results and Discussion

### 3.1. Thermal Properties of the PP/MWCNT Nanocomposites

**Figure 2:**
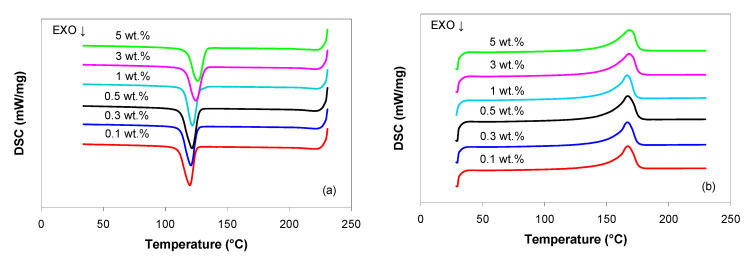
DSC cooling (**a**) and second heating (**b**) curves for the PP/MWCNT nanocomposites.

Figure 2 shows the DSC curves for the cooling and second heating scans (after removal of the thermal history due to processing–melt mixing conditions), while the thermal properties extracted from the DSC curves, such as crystallization and melting temperatures, ejection temperature, crystallization enthalpy and degree of crystallinity, are summarized in Table 1.

The DSC cooling scan (Figure 2a) displays a single crystallization peak, which shifts gradually to higher temperatures as the MWCNT loading increases, indicating that the crystallization process is facilitated in the presence of MWCNTs (i.e., the nucleation starts around CNTs) as reported by [27,36,41,43,50,55,60,61]. From Table 1 it can be seen that the ejection temperature (e.g., the temperature at which the molded part can be ejected from the mold without distortion) also increases with increasing MWCNT loading, indicating that cycle time can be reduced by the addition of MWCNTs as the molded part can be safely ejected at higher temperatures without compromising the quality of the part.

The melting peak temperature of PP/MWCNT nanocomposites is not significantly affected by the addition of MWCNTs. The DSC heating scans (Figure 2b) and Table 1 show that the melting peak temperature is about 167–168 °C.

With regard to the melting and crystallization enthalpies (Table 1), no dependency on the MWCNT loading could be identified, similarly with that observed for other PP/MWCNT nanocomposites [36,41,55,60]. The degree of crystallinity is influenced by the addition of MWCNTs; however, the trend is not consistent. In particular, a progressive increase in the crystallinity of PP/MWCNT nanocomposites between 1 and 5 wt.% is observed. This can be related to the dispersion of carbon nanotubes in nanocomposite, as a good dispersion of fillers hinders the growth of PP crystallites [34,36,41,50,55,60,61].

### 3.2. Melt Flow Index

The MFI of the PP/MWCNT nanocomposites is shown in Table 2. With the increase of MWCNT loading up to 0.5 wt.%, the MFI remains generally constant since nanotubes were relatively dispersed in the PP matrix. At 230 °C, the MFI of the PP/MWCNT nanocomposite with 0.1 wt.% was not significantly different from that of the neat PP (e.g., 25 g/10 min at 230 °C, 2.16 kg). When the MWCNT loading increased to a certain extent (>1 wt.%), the MFI significantly decreased with the increase of MWCNT wt.%. Since MFI is a measure of the flow property, the sharp decrease of MFI reflects the increase of melt shear viscosity as a result of CNT-CNT and CNT-polymer interactions [39,40,41,45,49].

### 3.3. Rheological Behavior of PP/MWCNT Nanocomposites

**Figure 3:**
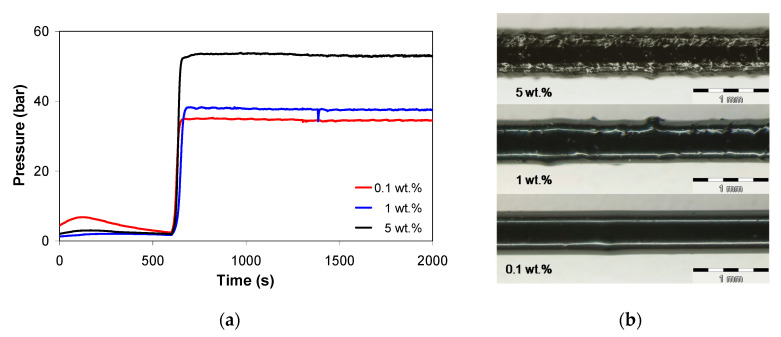
Pressure vs. time at apparent shear rate of 250 s^−1^ and 220 °C melt temperature (**a**) and extrudate filaments (**b**) for the PP/MWCNT nanocomposite with 0.1 wt.%, 1 wt.% and 5 wt.%.

To verify the stability of the PP/MWCNT nanocomposites during the rheological measurements and consequently during the manufacturing processes such as extrusion and injection molding, thermal stability tests were carried out. In general, if thermal degradation occurs in a polymer melt, the viscosity reduces as a result of decreasing molecular weight. Figure 3a shows the variation of pressure recorded during the extrusion of the PP/MWCNT nanocomposites with 0.1, 1, and 5 wt.% of MWCNTs at a constant shear rate and temperature of 250 s^−1^ and 220 °C, respectively. It can be seen that the pressure is constant during extrusion under constant shear rate, indicating very good stability of the nanocomposite melt during capillary extrusion. Moreover, the pressure increases with increasing MWCNT loading, due to the increase in nanocomposite melt viscosity. However, as MWCNT loading increases, the surface of the extrudate undergoes a transition from stable and glossy (up to 1 wt.%) to mat and stick-slip flow, most probably due to CNT agglomeration (5 wt.%), as shown in Figure 3b.

**Figure 4:**
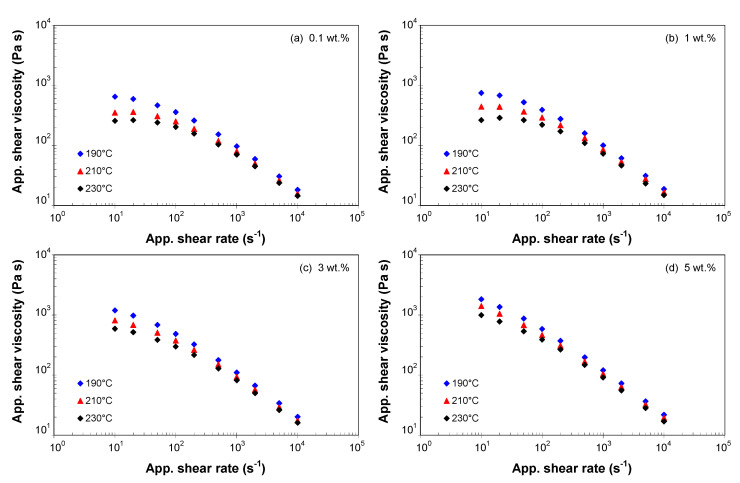
Apparent shear viscosity vs. apparent shear rate for the PP/MWCNT nanocomposite with (**a**) 0.1 wt.%, (**b**) 1 wt.%, (**c**) 3 wt.% and (**d**) 5 wt.% of MWCNTs (L/D = 30/1).

Figure 4 display the melt flow behavior of the PP/MWCNT nanocomposites, i.e., apparent melt shear viscosity versus apparent shear rate, at selected temperature and MWCNT loadings. At low MWCNT loadings (Figure 4a–c), the viscosity shows a Newtonian behavior (plateau) at low shear rates followed by a transition to the asymptotic power-law shear thinning for high shear rates.

At higher MWCNT loadings (Figure 4d), the PP/MWCNT nanocomposites behave like non-Newtonian fluids even at low shear rates since the CNT-CNT interactions dominate over the polymer-polymer interactions. However, at high shear rates, all nanocomposites showed shear-thinning behavior. High shear rates causes a breakdown in network structure and the CNTs become oriented in the flow direction, contributing to shear thinning behavior event at low loadings. In addition, the alignment of CNTs in the shearing direction causes the viscosity to decrease. Similar effect of CNTs has been reported in [29,39,43,45,51,62,63,64].

**Figure 5:**
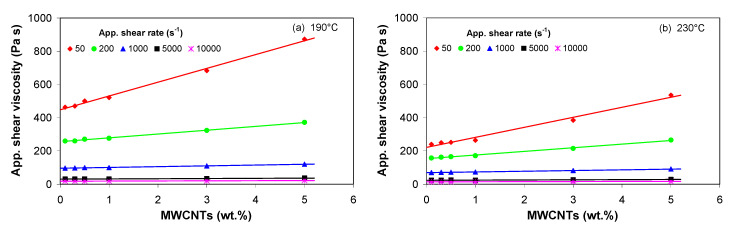
Apparent shear viscosity as a function of MWCNT loading at a shear rate of (**a**) 190 °C and (**b**) 230 °C.

Figure 5 shows the effect of MWCNTs on the melt shear viscosity of the PP/MWCNT nanocomposite. The viscosity of the PP/MWCNT nanocomposite remains unaffected on the addition of up to 1 wt.% of MWCNTs. However, on increasing the MWCNT loading up to 5 wt.%, the viscosity increases with increasing MWCNT loading, and the influence of MWCNTs is more significant at low shear rates, i.e., <200 s^−1^ (Figure 5). At higher shear rates, MWCNTs are more oriented and aligned in flow directions, which ultimately leads to alignment of the disengaged polymer melt chains and also provide more slippage on the surface of the polymer chains [65,66].

In general, MFI is used for quality control or for selecting an equivalent material in a material database [15,16]. However, the viscosity index (VI), which is a viscosity value at 1000 s^−1^, can be used instead of MFI since this shear rate value is in injection molding range and, always, is a data set of material data. Table 3 shows the variation of the viscosity index as a function of MWCNTs and temperature. It is observed that the viscosity index is independent of MWCNT loading up to 1 wt.%, while at higher loadings the viscosity index increases with increasing MWCNT wt.%.

### 3.4. Modeling of Melt Shear Viscosity

Melt viscosity is critical in predicting filling pattern and related characteristics, therefore viscosity models in which the viscosity is linked to the shear rate, temperature and pressure are required [13,14,15,16] for numerical modeling. As illustrated in Figure 4, the viscosity of the PP/MWCNT nanocomposites showed a general decrease by increasing shear rates from a constant level at low shear rate values to the asymptotic power-law shear thinning for high shear rate values. Therefore, to capture this behavior, the Cross model was adopted to describe the temperature, shear rate and MWCNTs dependency of viscosity. The Cross model is commonly used to model the flow of polymer melts during the filling stage of the injection molding process [13,15,16], according to

(4) $$\eta =\frac{\eta_{0}}{1+{\left(\frac{\eta_{0}}{\tau *}\dot{\gamma }\right)}^{1-n}},$$

where $\eta_{0}$ is the shear viscosity when the shear rate is approaching zero, τ* is the critical shear stress characterizing the transition from the Newtonian region to the pseudo-plastic region, and n is the shear-thinning index. The main advantage of the Cross model is that the model parameters have a physical meaning.

**Figure 6:**
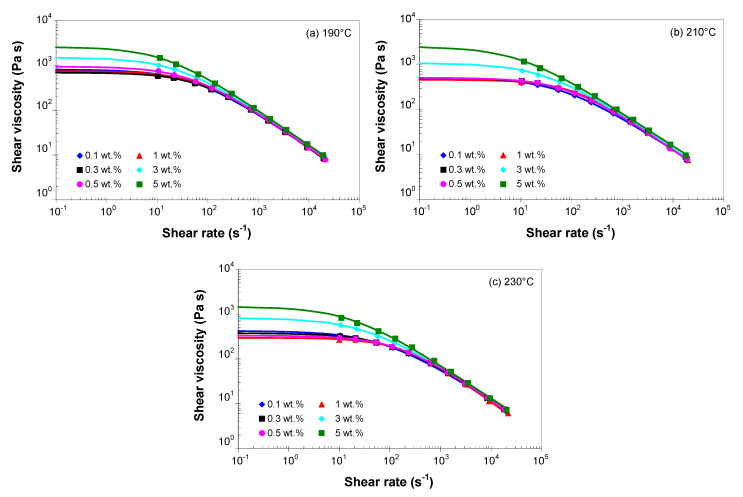
Cross model predictions (solid lines) and experimental data (symbols) for the PP/MWCNT nanocomposites at (**a**) 190 °C, (**b**) 210 °C and (**c**) 230 °C.

The Cross model was fitted to the (corrected) shear viscosity data using the WinRHEO II (Göttfert GmbH, Buchen, Germany) and the model parameters are listed in Supplementary Tables S1–S3. It should be noted that the $\eta_{0}$ values were obtained by extrapolation, since the smallest available shear rate was 10^−1^. Figure 6 shows a comparison between the viscosity curves predicted by the Cross model (Equation (4)) and experimental data for 190, 210, and 230 °C. It can be seen that the dependences of the melt shear viscosity on temperature, shear rate and MWCNT loading are well described by the Cross model, and the experimental data are very well predicted by this model.

Numerical simulation of the polymer flow requires the melt shear viscosity to be a single function of both shear rate and temperature [13,14,15,16,67]. Therefore, for each MWCNT loading, the experimental flow curves at different temperatures were shifted onto a master curve using the Arrhenius shifting factor [13,14,68]

(5) $$a_{T}=\exp\left[\frac{E_{a}}{R}\left(\frac{1}{T}-\frac{1}{T_{0}}\right)\right],$$

in which $E_{a}$ is the flow activation energy (J/mol), *R* is the universal gas constant (=8.314 J/mol K) and $T_{0}$ is the reference temperature (K).

The parameters for the master curves were determined by fitting the Cross model (Equation (4)) to the experimental master curves using the WinRHEO II (Göttfert GmbH, Buchen, Germany). Table 4 presents the model parameters at a reference temperature of 210 °C as well as the shifting factors and the activation energy for flow.

**Figure 7:**
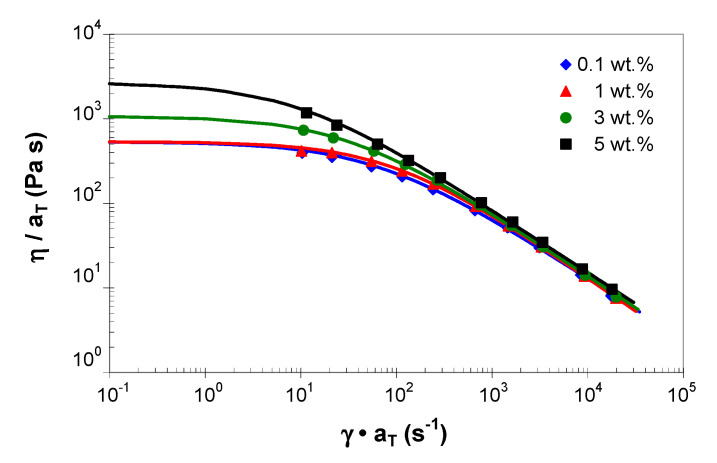
Comparison between the experimental shear viscosity master curves (symbols) at a reference temperature of 210 °C and the model predictions (solid curves).

Figure 7 shows the experimental master curves (symbols) and the fitted values using the Cross model (solid lines). For clarity, only several points along the experimental master curves were considered in Figure 7. In addition, the data for 0.3 and 0.5 wt.% were not shown because no statistically significant changes were observed in the rheological response of the PP/MWCNT nanocomposites with up to 1 wt.% of MWCNTs. As shown in Figure 7 and Table 4, the fitted viscosity curves are in very good agreement with the experimental mater curves.

Figure 7 indicates the tendency of the PP/MWCNT nanocomposite to change their rheological behavior at low shear rates with the addition of MWCNTs from a Newtonian to a solid like (non-Newtonian) behavior, due to the formation of a CNT network, which becomes denser with increasing MWCNT loading [47]. Furthermore, at shear rates higher than 500 s^−1^ all nanocomposites show a pronounced solid-like behavior (*n* in the range of 0.22 to 0.27). This result is related to the formation of CNT network-like structures due to increased interactions between CNTs as the nanotube loading increases [39,40,42,45,49,64]. Figure 7 also suggests that, at very high shear rates the viscosity curves could merge and become independent of the MWCNTs, which is an important advantage from the manufacturing point of view.

Another observation from Table 4 is that at low MWCNT loadings (1 wt.% and below), the values of $\eta_{0}$ parameter, as predicted by the Cross model, remain at the same level; however, there is a significant increase in the $\eta_{0}$ values at 3 wt.% and 5 wt.%. As stated before, the $\eta_{0}$ values were obtained by extrapolation and, therefore, should be interpreted with care as the smallest shear rate value in the experiment was 10 s^−1^ and the $\eta_{0}$ is recommended to be measured at 10^−3^ s^−1^ or less.

### 3.5. Specific Volume of PP/MWCNT Nanocomposites

**Figure 8:**
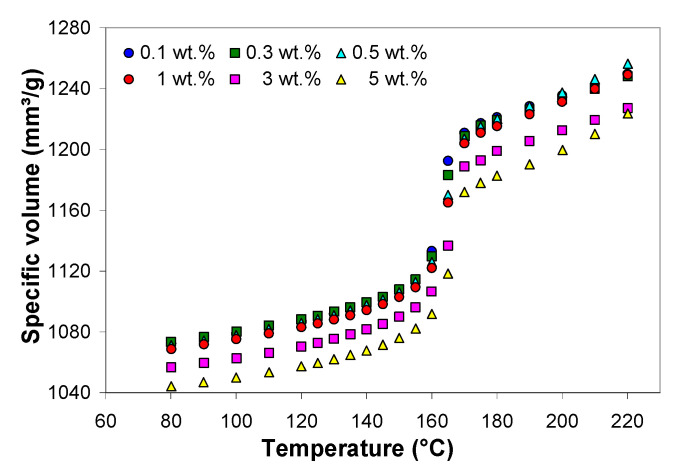
Effect of temperature and MWCNT wt.% on the specific volume of the PP/MWCNT nanocomposite at 500 bar.

Figure 8 shows the specific volume of the PP/MWCNT nanocomposites at 500 bar as a function of temperature and MWCNTs. It can be seen that the specific volume remains mostly unaffected by the addition of MWCNTs up to 1 wt.%. However, at higher loadings, the specific volume of the PP/MWCNT nanocomposite decreased with increasing MWCNT loadings. The reduction explains the effect of MWCNTs, which do not expand or contract as the temperature changes and counteract shrinkage effects due to molecular orientation. At 500 bar, the specific volume of the PP/MWCNT nanocomposites decreased by approx. 3 % as the MWCNT loading increased from 0.1 to 5 wt.%. Similar behavior was observed at other pressures.

The simulation of the polymer flow during the packing phase requires the *pVT* diagrams modeled by the modified 2-domain Tait equation [13,14,15,16] to determine the density as a function of the temperature and pressure. In addition, the shrinkage and warpage can be predicted, given the *pVT* diagrams and processing conditions [13,14,15,16,19,20].

**Figure 9:**
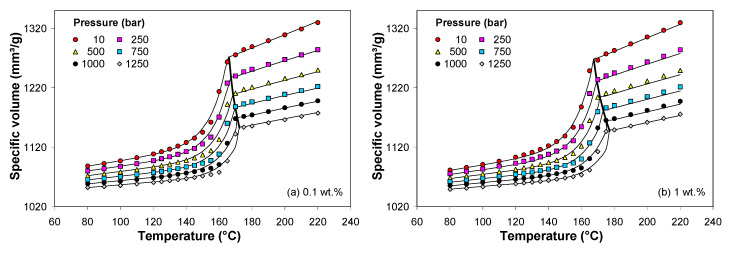

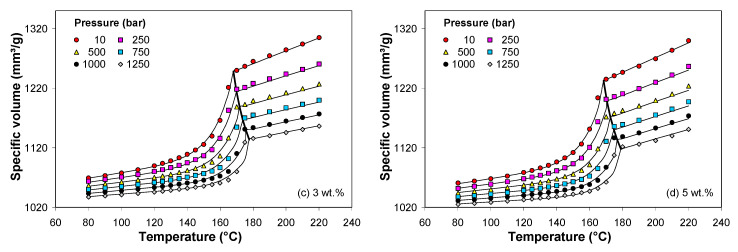
Comparison between the experimental *pVT* data (symbols) and 2-domain Tait model (solid lines) for PP/MWCNT nanocomposite with (**a**) 0.1 wt.%, (**b**) 1 wt.%, (**c**) 3 wt.% and (**d**) 5 wt.%.

Figure 9 shows the comparison between the experimental *pVT* data and the predictions based on the modified 2-domain Tait model presented in Section 1 of the Supplementary Materials. The fitting process was carried out using the WinRheo software (Göttfert GmbH, Buchen, Germany), and the model parameters are listed in the Supplementary Table S4. The Tait model appears to fit very well the experimental *pVT* data, as shown in Figure 9. The average absolute percentage errors between the experimental and predicted values were less than 0.3%.

**Figure 10:**
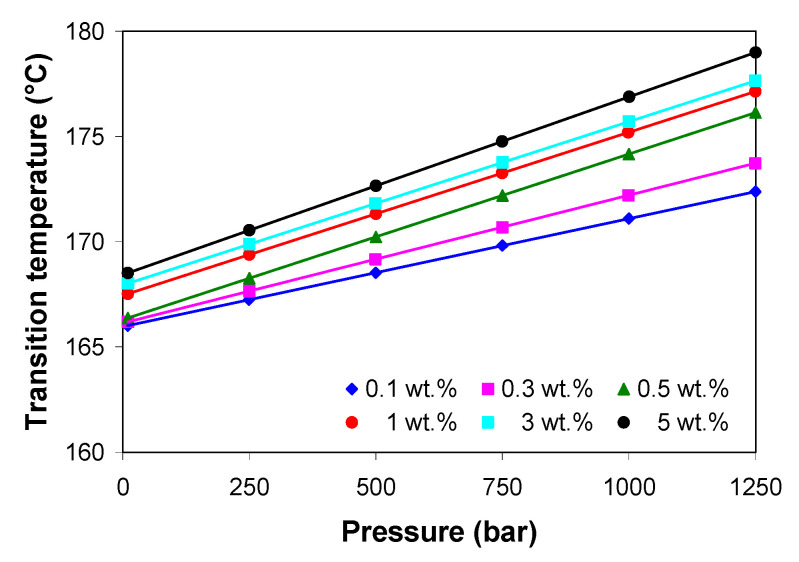
*pVT* transition temperature as a function of pressure and MWCNT loadings.

Figure 10 shows the transition temperature as a function of pressure and MWCNTs as predicted by the 2-domain Tait model. The transition temperature of the PP/MWCNT nanocomposites increased linearly with increasing pressure. The shift of the transition temperature to higher value indicates that, under pressure, the PP/MWCNT nanocomposites crystallizes at higher temperatures as compared with the DSC transition temperature, which varies between 120 to 126 °C (Table 1).

### 3.6. Density of PP/MWCNT Nanocomposites

**Figure 11:**
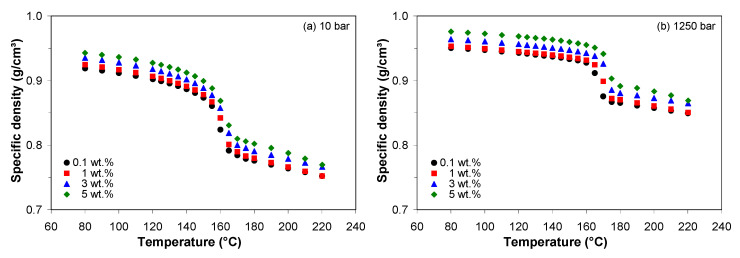
Density of the PP/MWCNT nanocomposites as a function of temperature and MWCNT loadings at pressures of (**a**) 10 bar and (**b**) 1250 bar.

Figure 11 shows the variation of the density calculated from the *pVT* data as a function of temperature and pressure for different MWCNT loadings. The data for 0.3 and 0.5 wt.% were not shown since there is no statistical difference between the density at 0.1, 0.3, and 0.5 wt.%. At constant pressure, the density of the PP/MWCNT nanocomposite decreases with increasing temperature and increases with increasing MWCNT loading. In addition to the temperature dependence, the density is dependent on the pressure, i.e., the specific density shifted to higher values with increasing pressure.

In simulation software, the density is generally given as a single value at the average processing temperature and zero pressure. The melt density (i.e., average value in the 200–220 °C temperature range) and solid density (i.e., average value in the 80–100 °C temperature range) at 0 bar pressure, as predicted by the Tait model, are summarized in Table 5. The bulk density (based on the Archimedes principle) of the PP/MWCNT nanocomposites is also listed in Table 5. The effect of MWCNTs on both solid and melt specific densities is similar–the density is independent of MWCNT loadings up to 1 wt.% and increases with further increase of MWCNT loading. Also, it is noted that the bulk density is smaller than the solid specific density and the trend varies with no specific pattern.

### 3.7. Thermal Conductivity of PP/MWCNT Nanocomposites

**Figure 12:**
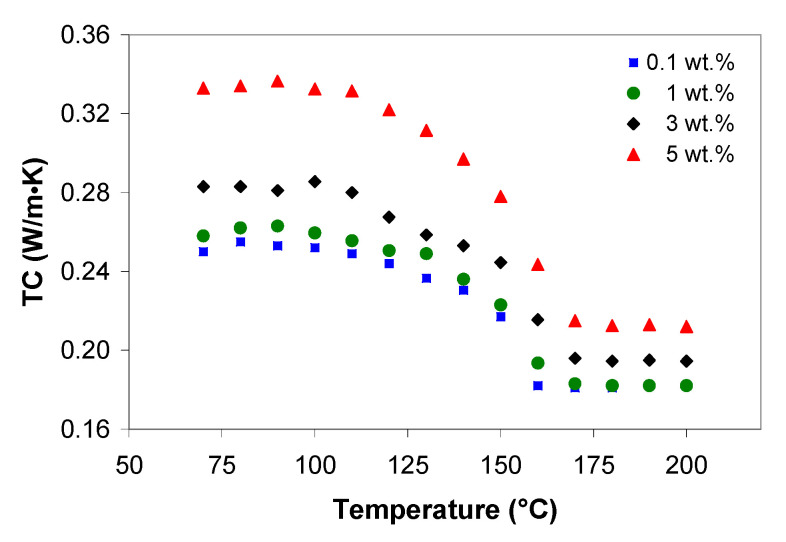
Effect of temperature and MWCNTs on thermal conductivity of the PP/MWCNT nanocomposites at 100 bar.

The influence of carbon nanotubes on the thermal conductivity of the PP/MWCNT nanocomposites as a function of temperature is shown in Figure 12 at 100 bar. It should be noted that, for nanotube loading up to 0.5 wt.%, the TC value was independent of MWCNTs and this observation correlates with the melt shear viscosity. Therefore, for clarity, in Figure 12, the curves for 0.3 and 0.5 wt.% were not shown. From Figure 12 it is evident that the TC displays three regions, corresponding to solid, transition and melt state: (i) a plateau up to 110 °C, (ii) a progressive (non-linear) decrease up to 160 °C, and (iii) a plateau beyond 160 °C. As can be seen, the TC is shifted towards higher values with increasing MWCNT loading. In particular, in solid state (70 °C), the TC for 0.1 wt.% is 0.25 W/m·K and increases up to 0.33 W/m·K for PP/MWCNT nanocomposite with 5 wt.%, corresponding to an increase of approx. 33%. In the melt state (170 °C), the TC for 0.1 wt.% is 0.182 W/m·K and increases up to 0.212 W/m·K for PP/MWCNT nanocomposite with 5 wt.%, corresponding to an increase of approx. 16%.

**Figure 13:**
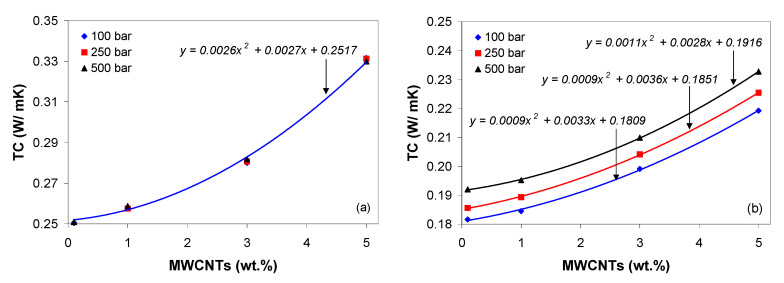
Effect of pressure on the thermal conductivity of the PP/MWCNT nanocomposites in (**a**) solid state and (**b**) melt state (The solid line relates to the quadratic model).

The second aspect investigated was the effect of pressure on the TC, which is very important for the injection molding process. In the solid and transition regions (Figure 13a), the increase of pressure does not improve the TC of PP/MWCNT nanocomposites (*p*-value = 0.48 > 0.05, *F*-stat = 0.83), whereas in the melt state (Figure 13b), the TC increases with increasing pressure and the effect is statistically significant (*p*-value = 0.00 < 0.05, *F*-stat = 0.229). This observation brings us to the conclusion that increasing the pressure helps improve CNT networks, increasing the contact between the CNTs [58,69]. Moreover, under pressure, the neighboring shell of MWCNTs could create new additional channels for phonons. The experimental data in Figure 13 were fitted to a quadratic model and the best fitted models with the coefficient of determination $R^{2}$ = 0.999 are given in Figure 13.

It should be noted that, the thermal conductivity data can be used to optimize the injection molding process (i.e., optimize the cycle times, avoid overheating, and reduce the scrap rates). Because of the increased TC, cycle times can be reduced as the parts will cool faster.

### 3.8. Stress–Strain Behavior of PP/MWCNT Nanocomposites

**Figure 14:**
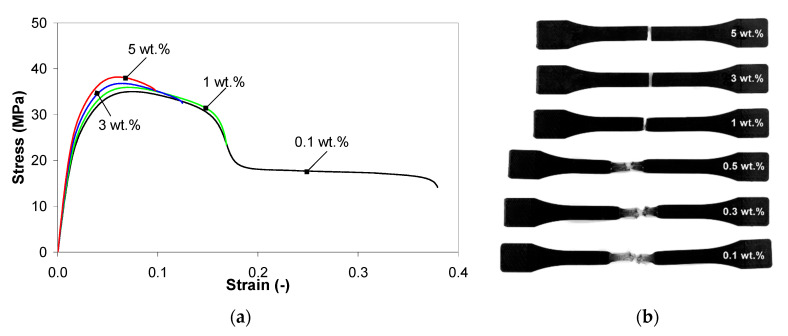
Stress-strain curves for the PP/MWCNT nanocomposites at 200 °C and 100 mm/min (**a**) and injection-molded samples after testing (**b**).

Figure 14 shows the representative stress-strain curves for the PP/MWCNT nanocomposites at 100 mm/min crosshead speed and 200 °C injection-molding temperature and the corresponding tested samples. In general, Figure 14a shows two types of stress-strain curves: (i) PP/MWCNT nanocomposites with lower loadings (0.5 wt.% and below) exhibit ductile behavior with yielding and necking/cold drawing with an elongation at break (~40%); (ii) PP/MWCNT nanocomposites with higher loadings exhibit yielding and then breaking after yielding. The fracture behavior of the PP/MWCNT nanocomposites was significantly affected by the addition of MWCNTs, as shown in Figure 14b.

**Figure 15:**
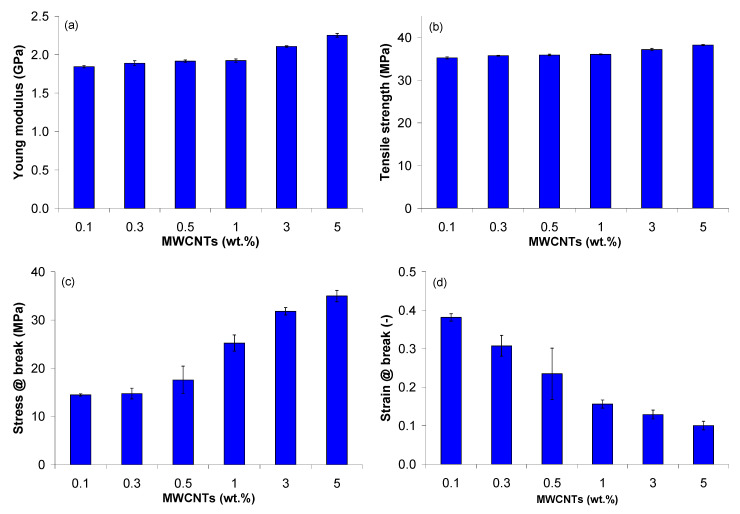
Mechanical properties of the PP/MWCNT nanocomposites: (**a**) Young modulus, (**b**) tensile strength, (**c**) stress at break, and (**d**) strain at break at 100 mm/min.

Mechanical properties, in terms of Young’s modulus, tensile strength, and stress and strain at break of PP/MWCNT nanocomposites are summarized in Figure 15 and Table 6. It should be noted that the Young’s modulus, tensile strength and strain at break of PP is 1.35 GPa, 32 MPa and >50%, respectively [54]. In general, the stiffness (tensile modulus and stress) of the nanocomposites increases with increasing MWCNT loadings at the expense of ductility (elongation at break). This reflects the reinforcement effect of MWCNTs on PP matrix by restricting the movement of polymer chains and bearing the force themselves [27,30,31,32,33,34,41,70]. For example, the Young modulus of the PP/MWCNT nanocomposite increased by approx. 40% with increasing MWCNT loading up to 1 wt.%, while a more important increase was recorded for higher loadings (+55% and +65% for 3 and 5 wt.%, respectively) as compared with the PP matrix.

The tensile strength was not significantly different for the PP/MWCNT nanocomposites with up to 1 wt.% of MWCNTs, but approx. 10% higher than that of the PP matrix, while the increase is more pronounced for higher MWCNT loadings (+16% and +20% for 3 and 5 wt.%, respectively) as compared with PP matrix, coherently to what elsewhere reported for PP/CNT nanocomposites [30,31,32,33,34,36,41]. When the content of MWCNTs was higher than 0.5 wt.%, the stress at break significantly increased with increasing MWCNT loading and reached the maximum value of about 35 MPa for the nanocomposite with 5 wt.%. The strain at break (elongation at break) progressively decreases with increasing MWCNT loading, as well known in the literature [30,31,32,33,34,35,36]. The higher the MWCNT content, the smaller the elongation at break is. The decrease in elongation at break indicates that the sample is more brittle and the presence of MWCNT agglomerations [30,31,32,33,34,35,36,70]. With increasing MWCNT loading, the nanotube-rich areas become bigger and tend to form clusters/agglomerations, and the nanocomposites would undergo degradation due to debonding and stress concentration around the MWCNT clusters/agglomerations [30,31,32,33,34,35,36,70].

Regarding the dependence of the mechanical responses of the PP/MWCNT nanocomposite on the applied strain rate (not shown here), it was observed that the tensile strength and stress at break increased with increasing crosshead speed, while the elongation at break decreased with increasing crosshead speed. The Young modulus also increased with increasing crosshead speed up to 100 mm/min, while with further increase of crosshead speed, the Young modulus decreased, although the modulus of all nanocomposites remained well above PP. This can be explained by the fact that at low crosshead speed, the sample is steadily deformed and the applied load can be transferred to MWCNTs embedded in PP matrix [41]. However, at higher crosshead speed, the effect is negative since the applied load cannot be transferred to MWCNTs.

The influence of the MWCNT wt.%, melt temperature and crosshead speed on the mechanical response of the PP/MWCNT nanocomposite was statistically examined based on the ANOVA, and the results are reported in Supplementary Tables S5–S8. The ANOVA for the Young modulus (Table S5) and tensile strength (Table S6) indicates that the MWCNT wt.%, temperature, crosshead speed and the interaction between MWCNTs and crosshead speed have a significant effect of the Young modulus and tensile strength (*p*-value < 0.05). On the other hand, the interaction between the temperature and MWCNTs and temperature and crosshead speed are not statistically significant. The ANOVA for stress at break (Table S7) and strain at break (Table S8) indicates that the MWCNTs and crosshead speed and the corresponding interaction are statistically significant, while the effect of temperature and the remaining interactions are not statistically significant (*p*-value > 0.05).

### 3.9. Electrical Properties of PP/MWCNT Nanocomposite

**Figure 16:**
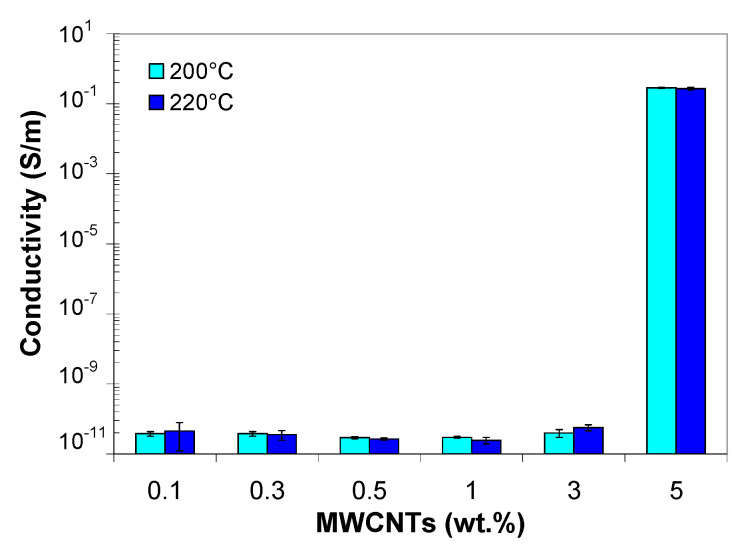
Effect of MWCNTs and injection molding temperature on the electrical conductivity of the PP/MWCNT nanocomposites.

Figure 16 shows the electrical conductivity of the PP/MWCNT nanocomposites measured in longitudinal direction, i.e., along the polymer flow direction. It can be seen that the electrical behavior of the PP/MWCNT nanocomposites changes from insulating to semiconducting range with increasing MWCNT loading. In addition, it is noted that the electrical conductivity of the PP/MWCNT nanocomposite is independent of the injection molding temperature.

In particular, the electrical conductivity is nearly independent of nanotube loading up to 3 wt.%, beyond which the conductivity increases with MWCNT loading. The electrical conductivity displays an abrupt increase from 10^−12^ S/m to 10^−1^ S/m, which is typical of the percolation phenomenon [71,72]. The 7-order increase indicates that the electrical percolation threshold in the PP/MWCNT nanocomposite is in the range of 3–5 wt.%, and a fully conductive network that spans the entire composite is formed at 5 wt.%. One of the reasons for the insulating nature of the PP/MWCNT nanocomposites up to 3 wt.% of MWCNTs is the absence of a continuous conductive path; as a result, the electrical conductivity is achieved by hopping of charge-carriers from one conductive site to another [71]. The high percolation threshold (between 3 and 5 wt.%) could be related to the crystallization process and the nucleating capability of CNTs in PP. Due to participating in the nucleation of PP crystals, CNTs could not concentrate and form conductive networks through an enhanced volume exclusion effect [46].

## 4. Conclusions

A comprehensive study was carried out to investigate the effect of MWCNTs on the physical, thermal, mechanical and electrical properties of the PP/MWCNT nanocomposites. The experimental results were complemented by theoretical calculations on the melt shear viscosity (Cross model) and specific volume (2-domain Tait model). The key observations can be concluded as follows:

- The melting and crystallization temperatures and degree of crystallinity of the PP/MWCNT nanocomposites are not significantly affected by the presence of the MWCNTs.
- With the addition of up to 5 wt.% of MWCNTs, the PP/MWCNT nanocomposite still remains as a non-Newtonian fluid and its shear thinning behavior at high shear rates render the nanocomposites processable by extrusion and injection molding.
- The specific volume of the PP/MWCNT nanocomposites decreases with increasing MWCNT loading, especially in the range of 1–5 wt.%, leading to better dimensional stability after melt processing.
- The thermal conductivity of the PP/MWCNT nanocomposite decreases with increasing temperature and increases with increasing MWCNT loading. In the melt state, the thermal conductivity increases with increasing pressure and the effect is statistically significant. The highest value of thermal conductivity (0.35 W/m K) was achieved in the solid state at 5 wt.% of MWCNTs.
- The tensile modulus, tensile strength and stress at break progressively increase with increasing MWCNT loading and the effect of reinforcement is more significant above 1 wt.% of MWCNTs. Furthermore, the elongation at break significantly decreases with increasing MWCNT loading.
- The PP/MWCNT nanocomposite is not electrically conductive up to 3 wt.%, whereas at MWCNT loading higher than 3 wt.%, due to the formation of a fully conductive network, the nanocomposite is semiconductive, having a conductivity of approx. 10^−1^ S/m.
- The Cross and the modified 2-domain Tait models successfully predicted the melt shear viscosity and specific volume as a function of MWCNTs, respectively.

In summary, the materials property data supplemented by the Cross and Tait models can be used for numerical simulation of manufacturing processes such as injection molding and extrusion to predict the PP/MWCNT nanocomposites behavior during the early stage of the design.

## Figures and Tables

**Table 1:** The DSC thermal properties for the PP/MWCNT nanocomposites.

MWCNTs (wt.%)	Cooling Scan			Second Heating Scan	
	$T_{c}$ (°C) Crystallization Temperature	$\Delta H_{c}$ (J/g) Crystallization Enthalpy	$T_{s}$ (°C) Ejection Temperature	$T_{m}$ (°C) Melting Temperature	χ (%) Crystallinity Degree
0.1	119.70	101.60	110.50	167.10	47.06
0.3	120.70	97.95	112.15	166.80	46.17
0.5	121.70	111.00	112.53	167.00	51.16
1	121.90	95.01	114.79	166.30	45.48
3	124.60	98.40	116.26	168.00	49.31
5	126.30	97.27	117.38	167.90	50.16

**Table 2:** Melt flow index for the PP/MWCNT nanocomposites.

MWCNTs (wt.%)	MFI (g/10 min)	
	190 (°C)	230 (°C)
0.1	10.91 ± 0.25	23.74 ± 0.92
0.3	10.41 ± 0.16	23.65 ± 0.51
0.5	10.00 ± 0.23	21.86 ± 0.56
1	9.20 ± 0.13	19.54 ± 0.68
3	5.45 ± 0.11	12.00 ± 0.28
5	2.28 ± 0.06	5.48 ± 0.15

**Table 3:** Viscosity index for the PP/MWCNT nanocomposites.

MWCNTs (wt.%)	VI (Pa·s)		
	190 (°C)	210 (°C)	230 (°C)
0.1	96.69	79.47	70.16
0.3	97.17	80.80	71.51
0.5	99.56	82.66	71.65
1	100.07	84.94	72.75
3	109.88	93.09	81.86
5	120.30	104.70	91.07

**Table 4:** Cross-Arrhenius master curve parameters at a reference temperature of 210 °C and the activation energy of viscous flow for the PP/MWCNT nanocomposites.

Parameters		MWCNTs (wt.%)					
		0.1	0.3	0.5	1	3	5
$\eta_{0}$ (Pa·s)		553.998	505.581	525.709	465.219	1104.245	2610.857
$\tau^{*}$ (Pa)		35,297.113	47,283.629	48,039.684	60,265.852	34,150.770	24,314.338
n		0.263	0.221	0.217	0.176	0.245	0.262
*R*^2^ Cross		0.995	0.999	0.998	0.998	0.997	0.999
$a_{T}$	190 °C	1.540	1.492	1.617	1.626	1.586	1.704
	200 °C	1.235	1.216	1.265	1.269	1.253	1.704
	210 °C	1.000	1.000	1.000	1.000	1.000	1.000
	220 °C	0.817	0.829	0.798	0.796	0.805	0.779
	230 °C	0.672	0.692	0.642	0.639	0.654	0.612
*E_a_* (J/mol)		40,151.755	37,191.234	44,717.904	45,230.321	42,880.345	49,569.519
*R*^2^ Arrhenius		0.979	0.994	0.990	0.994	0.993	0.996

**Table 5:** Density of the PP/MWCNT nanocomposites.

MWCNTs (wt.%)	Melt Density (g/cm^3^)	Solid Density (g/cm^3^)	Bulk Density (g/cm^3^)
0.1	0.756 ± 0.0053	0.915 ± 0.0032	0.884 ± 0.0024
0.3	0.757 ± 0.0053	0.916 ± 0.0031	0.883 ± 0.0026
0.5	0.756 ± 0.0053	0.918 ± 0.0032	0.877 ± 0.0035
1	0.759 ± 0.0054	0.921 ± 0.0032	0.861 ± 0.0028
3	0.772 ± 0.0054	0.931 ± 0.0033	0.888 ± 0.0018
5	0.778 ± 0.0061	0.940 ± 0.0031	0.883 ± 0.0035

**Table 6:** Mechanical properties of the PP/MWCNT at 100 mm/min and 200 °C.

MWCNTs (wt.%)	Young Modulus (GPa)	Tensile Strength (MPa)	Stress at Break (MPa)	Strain at Break (-)
0.1	1843.08 ± 16.06	35.23 ± 0.25	14.45 ± 0.24	0.38 ± 0.01
0.3	1887.44 ± 32.20	35.75 ± 0.11	14.74 ± 1.11	0.31 ± 0.03
0.5	1914.60 ± 16.54	35.90 ± 0.18	17.56 ± 2.84	0.23 ± 0.07
1	1922.14 ± 21.09	36.06 ± 0.10	25.21 ± 1.67	0.16 ± 0.01
3	2105.26 ± 9.40	37.18 ± 0.26	31.81 ± 0.76	0.13 ± 0.01
5	2251.56 ± 24.54	38.23 ± 0.13	34.97 ± 1.17	0.10 ± 0.01

## Data Availability

The data presented in this study are available in article or supplementary material.

## Supplementary Materials

The following are available online at https://www.mdpi.com/2073-4360/13/2/187/s1, 1. Modified 2-domain Tait equation; 2. Tables: Table S1. Cross model parameters at 190 °C, Table S2. Cross model parameters at 210 °C, Table S3. Cross model parameters at 230 °C, Table S4. Modified 2-domain Tait parameters for the PP/MWCNT nanocomposites, Table S5. Analysis of Variance for Young modulus, Table S6. Analysis of Variance for tensile strength, Table S7. Analysis of Variance for stress at break, Table S8. Analysis of Variance for strain at break.
