# Supplementary material for: Thermal, Rheological, Mechanical, and Electrical Properties of Polypropylene/Multi-Walled Carbon Nanotube Nanocomposites
Source: Polymers (Basel). 2021 Jan 7;13(2):187. doi: 10.3390/polym13020187 (PMC7825608; doi:10.3390/polym13020187)
Supplement: Supplementary file 1 [file polymers-13-00187-s001.pdf]

polymers

Supplementary Materials

Thermal, Rheological, Mechanical, and Electrical Properties of Polypropylene/Multi-Walled Carbon Nanotube Nanocomposites

Nicoleta-Violeta Stanciu, Felicia Stan *, Ionut-Laurentiu Sandu, Catalin Fetecau and Adriana-Madalina Turcanu

Center of Excellence Polymer Processing, Dunarea de Jos University of Galati, 47 Domneasca, 800 008 Galati, Romania; nicoleta.stanciu@ugal.ro (N.-V.S.); felicia.stan@ugal.ro (F.S.); laurentiu.sandu@ugal.ro (I.-L.S.); catalin.fetecau@ugal.ro (C.F.); madalina.constantinescu@ugal.ro (A.-M.T.)

* Correspondence: felicia.stan@ugal.ro

1.Modified 2-domain Tait equation

According to the modified 2-domain Tait equation, the relationship between the specific volume, pressure and temperature can be expressed by [1]

$$V(T,p) = V_0(T)\cdot\left[1 - C\cdot\ln\left(1+\frac{p}{B(T)}\right)\right] + V_t(T,p), \tag{S1}$$

in which

$$V_0(T) = \begin{cases} b_{1s} + b_{2s}\cdot(T-b_5), & T \le T_t(p) \\ b_{1m} + b_{2m}\cdot(T-b_5), & T > T_t(p) \end{cases}, \tag{S2}$$

$$B(T) = \begin{cases} b_{3s}\cdot\exp[-b_{4s}\cdot(T-b_5)], & T \le T_t(p) \\ b_{3m}\cdot\exp[-b_{4m}\cdot(T-b_5)], & T > T_t(p) \end{cases} \tag{S3}$$

$$V_t(T,p) = \begin{cases} b_7\cdot\exp[b_8\cdot(T-b_5) - b_9\cdot p], & T \le T_t(p) \\ 0, \quad T > T_t(p) \end{cases}, \tag{S4}$$

$$T_t(p) = b_5 + b_6\cdot p\,. \tag{S5}$$

where b_1 to b_9 are material constants, and T_t is the transition temperature between melt ("m") and solid ("s").

References

1. Chang, R.Y.; Chen, C.H.; Su, K.S. Modifying the Tait equation with cooling-rate effects to predict the pressure-volume-temperature behaviors of amorphous polymers: Modeling and experiments. *Polym. Eng. Sci.*, **1996**, *36(13)*, 1789-1795.

Citation: Stanciu, N.-V.; Stan, F.; Sandu, I.-L.; Fetecau, C.; Turcanu, A.-M. Thermal, Rheological, Mechanical, and Electrical Properties of Polypropylene/Multi-Walled Carbon Nanotube Nanocomposites. *Polymers* **2021**, *13*, 187. https://doi.org/10.3390/polym13020187

Academic Editor: Jea Uk Lee
Received: 16 December 2020
Accepted: 02 January 2021
Published: 07 January 2021

Publisher's Note: MDPI stays neutral with regard to jurisdictional claims in published maps and institutional affiliations.

Copyright: © 2021 by the authors. Licensee MDPI, Basel, Switzerland. This article is an open access article distributed under the terms and conditions of the Creative Commons Attribution (CC BY) license (http://creativecommons.org/licenses/by/4.0/).

2. Tables

Table S1. Cross model parameters at 190 °C.

Parameters.	MWCNTs (wt.%)					
	0.1	0.3	0.5	1	3	5
η_0 (Pa·s)	810.652	691.540	945.898	747.507	1503.012	2600.283
τ^* (Pa)	43,924.715	56,379.652	47,110.094	57,940.332	42,273.320	39,532.391
n	0.233	0.197	0.216	0.188	0.224	0.222
R^2	1.000	1.000	1.000	1.000	1.000	1.000

Table S2. Cross model parameters at 210 °C.

Parameters	MWCNTs (wt.%)					
	0.1	0.3	0.5	1	3	5
η_0 (Pa·s)	508.785	504.639	513.085	464.473	1086.767	2551.714
τ^* (Pa)	33879.566	47437.984	47038.563	60569.996	33727.516	24206.486
n	0.271	0.222	0.218	0.177	0.246	0.268
R^2	1.000	1.000	1.000	0.999	1.000	1.000

Table S3. Cross model parameters at 230 °C.

Parameters	MWCNTs (wt.%)					
	0.1	0.3	0.5	1	3	5
η_0 (Pa·s)	422.074	374.814	330.881	296.408	820.807	1473.837
τ^* (Pa)	30;374.111	40;667.691	51;935.113	61;123.297	28;159.422	25;729.813
n	0.284	0.246	0.208	0.160	0.267	0.259
R^2	0.999	1.000	1.000	0.999	1.000	1.000

Table S4. Modified 2-domain Tait parameters for the PP/MWCNT nanocomposites.

Parameters	MWCNTs (wt.%)					
	0.1	0.3	0.5	1	3	5
b_{1s} (mm³/g)	1.1279×10³	1.1256×10³	1.1234×10³	1.1211×10³	1.1087×10³	1.0974×10³
b_{2s} (mm³/g °C)	0.4585	0.4505	0.4533	0.4552	0.4482	0.4243
b_{3s} (bar)	1.5889×10³	1.7192×10³	1.6874×10³	1.7097×10³	1.6684×10³	1.4761×10³
b_{4s} (1/°C)	0.0064	0.0069	0.0073	0.0072	0.0074	0.0075
b_{1m} (mm³/g)	1.2731×10³	1.2713×10³	1.2734×10³	1.2497×10³	1.2497×10³	1.2344×10³
b_{2m} (mm³/g °C)	1.1299	1.1189	1.1369	1.1389	1.0960	1.2407
b_{3m} (bar)	6.3267×10²	6.4173×10²	6.0147×10²	5.9844×10²	6.3468×10²	5.9190×10²
b_{4m} (1/°C)	0.0057	0.0061	0.0050	0.0049	0.0054	0.0038
b_5 (°C)	165.9642	166.1286	166.2931	167.4445	167.9380	168.4314
b_6 (°C/bar)	0.0051	0.0061	0.0079	0.0078	0.0078	0.0084
b_7 (mm³/g)	154.3777	148.8329	149.7084	146.3524	136.7220	123.2979
b_8 (1/°C)	0.0979	0.1079	0.0922	0.0922	0.0880	0.0882
b_9 (1/bar)	0.0012	0.0013	0.0015	0.0014	0.0014	0.0013

Table S5. Analysis of Variance for Young modulus.

Source	DF	Seq SS	Adj SS	Adj MS	F	P
MWCNTs (wt.%)	5	638871	638871	127774	283.16	0.000
Injection molding temperature (°C)	1	7553	7553	7553	16.74	0.002
Crosshead speed (m/min)	2	59755	59755	29878	66.21	0.000
MWCNTs (wt.%)×Temperature (°C)	5	2231	2231	446	0.99	0.471
MWCNTs (wt.%)×Crosshead speed (m/min)	10	15678	15678	1568	3.47	0.031

Temperature (°C)×Crosshead speed (m/min)	2	479	479	240	0.53	0.604
Residual Error	10	4512	4512	451		
Total	35	729080				

Table S6. Analysis of Variance for tensile strength.

Source	DF	Seq SS	Adj SS	Adj MS	F	P
MWCNTs (wt.%)	5	36.638	36.638	7.3277	334.45	0.000
Injection molding temperature (°C)	1	0.623	0.623	0.6229	28.43	0.000
Crosshead speed (m/min)	2	194.403	194.403	97.2015	4436.54	0.000
MWCNTs (wt.%) ×Temperature (°C)	5	0.077	0.077	0.0154	0.70	0.635
MWCNTs (wt.%) ×Crosshead speed (m/min)	10	1.769	1.769	0.1769	8.07	0.001
Temperature (°C) ×Crosshead speed (m/min)	2	0.111	0.111	0.0554	2.53	0.129
Residual Error	10	0.219	0.219	0.0219		
Total	35	233.840				

Table S7. Analysis of Variance for stress at break.

Source	DF	Seq SS	Adj SS	Adj MS	F	P
MWCNTs (wt.%)	5	1256.77	1256.77	251.355	124.23	0.000
Injection molding temperature (°C)	1	0.38	0.38	0.383	0.19	0.673
Crosshead speed (m/min)	2	1563.76	1563.76	781.881	386.45	0.000
MWCNTs (wt.%) ×Temperature (°C)	5	3.09	3.09	0.618	0.31	0.899
MWCNTs (wt.%) ×Crosshead speed (m/min)	10	305.98	305.98	30.598	15.12	0.000
Temperature (°C) ×Crosshead speed (m/min)	2	0.86	0.86	0.429	0.21	0.812
Residual Error	10	20.23	20.23	2.023		
Total	35	3151.08				

Table S8. Analysis of Variance for strain at break.

Source	DF	Seq SS	Adj SS	Adj MS	F	P
MWCNTs (wt.%)	5	8.1921	8.1921	1.63841	44.32	0.000
Temperature (°C)	1	0.0506	0.0506	0.05064	1.37	0.269
Crosshead speed (m/min)	2	19.2763	19.2763	9.63816	260.73	0.000
MWCNTs (wt.%) ×Temperature (°C)	5	0.2198	0.2198	0.04397	1.19	0.380
MWCNTs (wt.%) ×Crosshead speed (m/min)	10	12.6705	12.6705	1.26705	34.28	0.000
Temperature (°C) ×Crosshead speed (m/min)	2	0.0566	0.0566	0.02831	0.77	0.490
Residual Error	10	0.3697	0.3697	0.03697		
Total	35	40.8356				
